# Comparison of temporal fine structure sensitivity and concurrent vowel perception between children with and without reading disability

**DOI:** 10.12688/f1000research.21544.2

**Published:** 2021-12-15

**Authors:** Arivudainambi Pitchaimuthu, Eshwari Ananth, Jayashree S Bhat, Somashekara Haralakatta Shivananjappa

**Affiliations:** 1 Department of Audiology & Speech Language Pathology, Kasturba Medical College, Mangalore, Manipal Academy of Higher Education (MAHE), Manipal, Karnataka, 575001, India

**Keywords:** Reading deficits, Temporal fine structure sensitivity, Concurrent vowel perception, auditory stream segregation.

## Abstract

**Background:** Children with reading disabilities (RD) exhibit difficulty in perceiving speech in background noise due to poor auditory stream segregation. There is a dearth of literature on measures of temporal fine structure sensitivity (TFS) and concurrent vowel perception abilities to assess auditory stream segregation in children with reading disabilities. Hence the present study compared temporal fine structure sensitivity (TFS) and concurrent vowel perception abilities between children with and without reading deficits.

**Method:** The present research consisted of a total number of 30 participants, 15 children with reading disabilities (RD) and fifteen typically developing (TD) children within the age range of 7-14 years and were designated as Group 1 and Group 2 respectively. Both groups were matched for age, grade, and classroom curricular instructions. The groups were evaluated for TFS and concurrent vowel perception abilities and the performance was compared using independent ‘t’ test and repeated measure ANOVA respectively.

**Results:** Results revealed that the children with RD performed significantly (p < 0.001) poorer than TD children on both TFS and concurrent vowel identification task. On concurrent vowel identification tasks, there was no significant interaction found between reading ability and F0 difference suggesting that the trend was similar in both the groups.

**Conclusion:** The study concludes that the children with RD show poor temporal fine structure sensitivity and concurrent vowel identification scores compared to age and grade matched TD children owing to poor auditory stream segregation in children with RD.

## Introduction

Reading is defined as a cognitive process by which one derives meaning from printed symbols ^1^. Children’s reading ability relies on the integration of rudimentary perceptual abilities with higher-order linguistic function ^2^. According to the simple view, the reading ability can result only from the combination of word decoding and reading comprehension. In contrast, the reading disability can result from either deficit only in word decoding, reading comprehension, or both ^3^. On the other hand, developmental dyslexia describes children with RD who demonstrate difficulty with single word reading accuracy or fluency in the context of intact cognitive skills and adequate educational opportunity ^4^. It is estimated to be impaired in 5 to 17% of the school-going population and observed as the most common neurodevelopmental disorder in children ^5^. An epidemiological study conducted in a south Indian city reported a 13.7% prevalence of dyslexia among school going children ^6^. In another study, the prevalence of specific learning disabilities was 15.17% in sampled children, whereas 12.5%, 11.2%, and 10.5% had dysgraphia, dyslexia, and dyscalculia respectively ^7^. Children with reading disabilities (RD) demonstrate difficulty learning to read and spell, despite having adequate intelligence and conventional instruction. Although the pathophysiology of RD is unknown, some hypothesize that RD emerges due to the deficits in encoding, representing, and processing phonological information ^8– 10^. In other words, children with RD show difficulties in acquiring the ability to relate language-specific written codes such as letters to corresponding spoken codes such as phonemes, resulting in word decoding deficits, which are the most palpable impairment in the majority of children with RD ^11– 13^. The processing of phonological information is determined by crucial aspects of auditory speech perception. Perceiving and interpreting auditory information is one of the factors that affect language acquisition and academic performance in children with normal hearing ability. Hence, the auditory perceptual abilities are important for reading as well as for exploring different phonological features. Tallal ^14– 16^ reported that receptive language is processed via the auditory system and the deficits in auditory perceptual skills result in impaired phonology, leading to difficulty in reading.

Auditory processing plays a significant role in the acquisition of oral-aural language in children. Accurate extraction of spectral (frequency information) and temporal (timing) cues from the speech is essential for the meaningful representation of phonological cues at higher levels. The auditory processing deficit that disrupts the coding of these acoustic cues may lead to inadequate representation of speech sounds. Studies have been done to identify the underlying cause of dyslexia, which gave rise to many theories. An influential hypothesis by Tallal, Merzenich, Miller, & Jenkins ^17^ states that any deviant recognition of fast acoustic events in speech hampers the normal development of phonological systems. Various research findings support this hypothesis and indicate that poor readers exhibit difficulty in the recognition of rapid acoustic events in speech and non-speech signals ^16– 18^. Tallal ^11^ reported that children with RD have difficulty in perceiving auditory stimuli with short duration and sounds that occur in rapid series. Such a deficit in auditory processing may compromise the temporal analysis of speech at the phoneme level, thus affecting the development of phoneme representations. These limitations pose specific challenges to children with RD to acquire both oral as well as written language.

Auditory processing deficit in children with RD also manifests as speech perception difficulties ^19^. For example, Hazan ^20^ reported that 30% of children with dyslexia exhibited speech perception deficits. Similarly, Manis *et al.* ^21^ also found that 28% of individuals with dyslexia showed a deficit in categorical perception for the voicing continuum. Tasks employed to assess categorical perception typically utilize optimal listening conditions. In such conditions, specific phoneme identification deficits would be compensated by the higher functions ^21– 23^. That may be the possible reason for the prevalence of categorical perception deficits only in a small set of the population with dyslexia. However, when the perceptual ability was assessed in less than optimal listening conditions, the majority of the individuals with dyslexia exhibited perceptual deficits. Blomert and Mitterer ^24^ reported that individuals with dyslexia exhibit considerable difficulty in the perception of synthetic speech when compared to natural speech. Similarly, individuals with dyslexia demonstrate more difficulty in perceiving speech in the presence of noise despite having good perception abilities in quiet conditions ^12^.

Generally, the difficulties to perceive speech in the presence of background noise are one of the common complaints of children with auditory processing deficits ^19, 25– 27^. The ability to perceive speech in the presence of background noise necessitates the auditory system to segregate the background noise from the target speech signal, and the process is referred to as auditory stream segregation ^28^. The critical acoustic cue that helps in auditory stream segregation/formation is a temporal fine structure (TFS). TFS helps to segregate the target speech and background noise into two separate streams ^29^. There is a dearth of research that investigates auditory stream segregation abilities among children with RD. Few attempts have been made to investigate the perception of TFS by individuals with RD ^30^. In this study, iterated rippled noise (IRN) pitch perception was considered as the measure of TFS sensitivity. However, recent investigations have shown that IRN pitch perception does not reflect the TFS sensitivity as spectral modulations mediate the perception of IRN pitch. Hence, TSF sensitivity in children with RD needs to be investigated using a validated method. Apart from IRN pitch perception, the TFS1 ^31, 32^, TFS-LF ^32^, TFS-AF ^33^, and frequency modulation detection limen (FMDL) test ^34^ can be used to measure the TFS sensitivity. The TFS1 method was adopted in the present study to assess the TFS sensitivity in children with RD. The TFS1 method adopted in the current study utilizes a complex tone discrimination task to estimate the TFS sensitivity. Additionally, auditory stream segregation can be studied reliably by using the most commonly considered paradigm, such as concurrent vowel identification paradigm ^35, 36^, which has high relevance to the perception of speech in the presence of noise. Hence, the present study compares the performance between children with reading disabilities and typically developing children on TFS sensitivity and concurrent vowel identification tasks.

## Method

### Study background

The present study employed a cross-sectional study design and a non-random convenient method of sampling to recruit the participants. The study was carried out in a school located in Mangalore, a city of Dakshina Kannada District of coastal Karnataka. The study was conducted between December 2018 and January 2019. The study proceeding was approved by the Institutional Ethics Committee of Kasturba Medical College, Mangalore. To avoid the variability in curricular material and method of teaching, all the participants were selected from a single school with the instructional medium being the Kannada language and the school curriculum affiliated to the Karnataka state board. Initially, permission from the school administrative authority was obtained to conduct the study on children within the school premises. Later, the parents of participants were informed about the nature of the study, and written consent was obtained before initiating the study.

### Participants

The present research consisted of a total number of 30 participants, 15 children with RD, and 15 TD children, within the age range of 7–14 years, who were designated as Group 1 and Group 2, respectively. The 15 children with RD were pooled from Grade II (N=1), Grade III (N=1), Grade IV (N=2), Grade V (N=2), Grade VI (N=5), and Grade VII (N=4) within the age range of 7.6 to 8.6 years, 8.7 to 9.6 years, 9.7 to 10.6 years, 10.7 to 11.6 years, 11.7 to 12.6 years and 12.7 to 13.6 years respectively. A similar pool of TD children was selected to match the age and Grade of RD children. The Linguistic Profile Test in Kannada ^37^ was used to ascertain age-appropriate language development in both groups. The Reading Acquisition Profile in Kannada ^38^ was administered to determine the reading abilities of children from both groups. The participants with the performance falling below 2SD of mean scores of TD criteria were classified as RD. All participants had hearing sensitivity within normal limits as their hearing thresholds ≤25 dBHL at audiometric octave frequencies (250 to 8000 Hz). None of the participants had gross otologic and neurologic deficits. Initially, the class teachers were requested to rate the children’s performance based on their perception of reading, writing, spelling, arithmetic skills, oral language comprehension, and expression skills according to the Grade level and in comparison with all other children in the classroom in every domain of language and literacy skills using a 5-point Likert rating scale (0 = poor, 1 = below average, 2 = good, 3 = very good, 4 = outstanding). Parents of the selected children were explained about the study and written consent was obtained from them. The children who were rated as poor and below average were further assessed for language and reading assessment to confirm the presence of RD based on the study criteria.

### Instrumentation and procedure

The following section explains the signal processing, instrumentation, and administration procedures of TFS sensitivity and concurrent vowel identification task measures. Each procedure was performed once per child.

### TFS Sensitivity

***Signal processing.*** A complex tone discrimination task as in the TFS1 was adopted in the current study. A complex harmonic tone (H) with the fundamental frequency (F0) of 100 Hz was created with a sampling frequency of 44,100 Hz. Inharmonic complex tone (I) was created by adding a fixed frequency difference (∆f) to the harmonic complex tone. Participant’s TFS sensitivity was estimated as minimum ∆f that is required to discriminate ‘H’ and ‘I.’ All the components of both harmonic and inharmonic complex tones had equal amplitudes. Partials of the ‘H’ and ‘I’ had random phases. Spectral content between 9 ^th^ and 13 ^th^ harmonics was retained while all others were filtered out using a 5 ^th^ order digital butter-worth filter to reduce cues related to the difference in excitation patterns. The participant’s ability to discriminate between harmonic and inharmonic complex was assessed as a function of ∆f. Pink noise was presented along with H and I tones to avoid the audibility of combination tones.

***Procedure.*** The stimuli were presented from a laptop (TOSHIBA, with intel core i5 processor) routed through the sound card (creative sound blaster X-fi USB 2, 24-bit digital-to-analog converter) and given through circum-aural stereo headphones (Sennheiser HD280Pro). To estimate TFS sensitivity, a transformed up-down procedure (2-down 1-up) with two intervals, two alternatives forced-choice (2IAFC) task was used. The stimuli were designed and presented through the laptop. For the TFS task, participants were presented with two intervals, target, and non-target interval. The non-target interval consisted of four harmonic complex tones in sequence with the inter-stimulus interval of 100 milliseconds (HHHH). In the target interval, two harmonic and two inharmonic complex tones were presented alternately with the inter-stimulus interval of 100 milliseconds (HIHI). The participant’s task was to identify the interval containing the HIHI pattern. The target and non-target intervals were presented in a random sequence, and the gap between the intervals was 500 milliseconds. Participants responded by clicking on the push buttons appearing on the computer screen. The participants pressed the push-button “1” if the target was present in the first interval, and pressed push-button “2” if the target was present in the second interval. The test started with ∆f of 50 Hz, and the ∆f was varied adaptively in a 2 Hz step size. For every two consecutive positive responses, ∆f was reduced by 2 Hz, and for every single negative response, ∆f was increased by 2 Hz. Midpoints of the last six reversals from the total eight reversals were averaged to estimate the thresholds. If the ∆f values exceeded 50 Hz, then 40 trials were presented at ∆f of 50 Hz, and total correct response scores were obtained.

### Concurrent vowel identification

***Signal processing.*** Five steady-state vowels /a/, /i/, /e/, /u/, /o/ was synthesized at the sampling rate of 44,100 Hz, using Klatt synthesizer. Each vowel was synthesized with F0 of 200 Hz. All five vowels were synthesized again with different F0 which was corresponding to 1, 2, and 4 semitones increase from base F0. So, this resulted in a total of 20 vowels. Each vowel had a duration of 270 msec with 20 ms raised cosine onset/offset ramps. Concurrent vowel pairs were created by pairing vowels with each other across different vowels and F0 conditions (5 vowels and 4 F0 conditions). The same vowel was never present in a pair, even if the F0 was different. This pairing resulted in combinations of vowels with the F0 difference of 0, 1, 2, and 4 semitones between them.

***Procedure.*** The stimuli were presented using the same instruments indicated for the measurement of TFS sensitivity. Stimuli were presented at the most comfortable level (MCL) as set by the participant before the test. The participants were asked to identify both the vowels that occurred during each presentation. Participants responded on a forced-choice paradigm where a response box consisting of all the five vowels appeared on the screen. Participants were instructed to respond by clicking on the appropriate buttons. Feedback was not provided during the session. Every vowel pair was presented 20 times. Percentage of correct identification of both vowels (double correct scores) and the percentage of correct identification of at least one vowel was calculated (single correct scores). Single correct scores were considered for further analysis as most of the children could not identify both the vowels.

### Statistical analysis

The results of the present study were analyzed using SPSS v17.0 statistical analysis software. The performance of children with RD on the TFS sensitivity test was compared with the performance of TD children using a parametric independent ‘t’ test. Similarly, the performance of children with RD on concurrent vowel identification tasks was compared with TD children using the parametric repeated measure ANOVA with subsequent post-hoc pair-wise comparison using Bonferroni’s test.

## Results

The present study aimed to investigate the auditory stream segregation abilities of children with RD in comparison with TD using complex discrimination as in TFS1 and concurrent vowel identification tasks. Underlying data from each participant is available at Harvard Dataverse.

### TFS sensitivity

**Figure 1.**
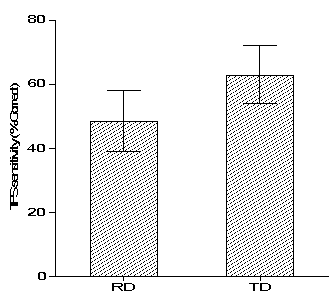
Bar graph depicting mean and standard deviation scores of temporal fine structure sensitivity in children with reading disability (RD) and typically developing (TD) children.

In the current study, the TFS sensitivity was assessed as the maximum ∆f that is required to differentiate harmonic and inharmonic complex tones. However, in most of the participants, the ∆f value exceeded 50% of the F0. Hence, the percentage of correct responses for differentiating harmonic and in-harmonic was measured for the ∆f value of 50% of the F0. The percentage of correct scores in both the RD (w = 0.90, p = 0.10) and TD (w = 0.91, p = 0.13) groups was normally distributed. A parametric independent sample t-test was done to investigate whether the percentage of correct response for differentiating harmonic and in-harmonic complex tones of children with RD was different from TD children. The analysis revealed that there was a significant difference in the percentage of the correct score between the children with RD and TD children (t28 = -4.31, p < 0.001). The percentage of the correct score of children with RD was significantly less than that of the TD children. This result suggests that children with RD have poorer TFS sensitivity than TD children. The mean and standard deviation of the percentage of correct scores for differentiating the harmonic and inharmonic complex tone is depicted in Figure 1.

### Concurrent vowel identification

**Figure 2.**
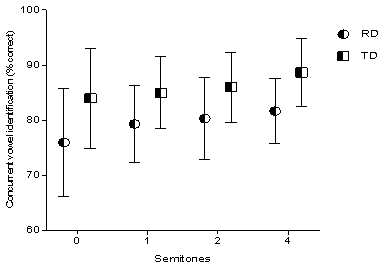
Plot graph depicting the concurrent vowel identification scores at different semitones for children with reading disability (RD) and typically developing (TD) children.

In the current study, the concurrent vowel identification task was used as a measure to assess the stream segregation ability. The children’s ability to correctly identify the two concurrently presented vowels was measured as a function of the F0. Since the children could not identify both the vowels, single correct scores were considered. Total correct scores for each F0 difference condition were measured, and these scores were compared between children with RD and TD children. Repeated measure ANOVA was done to investigate the main effect of reading ability (RD and TD) and F0 difference (0, 1, 2, and 4 semitone difference) on concurrent vowel identification. For the analysis, reading ability served as the between-subject factor, and the F0 difference served as a within-subject variable. The analysis revealed a significant main effect of the F0 difference on vowel identification (F (3,84) = 3.302, p=0.02). Effect of reading ability on concurrent vowel identification was also significant (F (1,28) = 13.84, p<0.001). Overall concurrent vowel identification scores of children with RD were significantly poorer than TD children. There was no significant interaction found between reading ability and F0 difference (F (3,84) = 0.23, p=0.88), suggesting that the trend was similar in both groups. To investigate the main effect of reading ability on CVI at each F0 difference condition, separate independent sample ‘t’ tests were performed. The results revealed that, reading ability had the significant main effect on CVI at 0 semitone (t28=-2.309, p=0.029), 1 semitone (t28=-2.283, p=0.030), 2 semitones (t28=-2.249, p=0.033) and, 4 semitones (t28=-3.197, p=0.003) f0 difference conditions. The mean and standard deviation of the consonant vowel identification at different semitones for both the group is depicted in Figure 2.

## Discussion

The present study investigated the auditory stream segregation abilities of children with RD in comparison with TD using TFS1 and concurrent vowel identification tasks. The TFS sensitivity of children with RD and TD was assessed using the TFS1 test ^31^. Statistical analysis revealed that children with RD have significantly poor TFS sensitivity when compared to TD children. In the human auditory system, TFS is represented by synchronous neural discharge, where the TFS information depends on the phase-locking to individual cycles of the stimulus carrier waveform (Moore, 2008). Some electrophysiological evidence has indicated a weak phase-locking ability in individuals with a learning disability ^39^. Frequency following response for speech stimulus has shown reduced phase locking to speech cues such as first formant frequency in individuals with a learning disability ^40^. Delay in neural timing leading to poor phase locking in children with a learning disability has also been demonstrated in various other studies ^40– 44^. In the current study, TFS sensitivity was assessed for the ability to discriminate the harmonic and in-harmonic complex tone as the function ∆f, wherein, ∆f used was 50% of F0 of complex harmonic tone. For such difference, the phase-locking mediates the discrimination ability ^31^. Reduced phase-locking ability present in children with RD could be the reason for the poor TFS sensitivity observed in the current study. The poor TFS sensitivity in children with RD supports the claim that the reading disorders may result from the auditory perceptual deficits in processing rapid auditory sequences ^16^. The poor efficiency in encoding vital acoustic and temporal features in the rapid auditory stream of speech can be the reason for abnormal phonological representation of speech sounds, which are crucial for reading acquisition in children with reading disability ^45– 47^. One important observation in the current study was that most children found it difficult to perform the discrimination task conducted in the current study. Hence future studies may consider utilizing children-friendly paradigms or modifying the procedures to suit the school age children. 

In the current study, concurrent vowel identification was used as a measure of auditory stream segregation. Individuals were presented with concurrent vowel (two vowels presented simultaneously) pairs, and their ability to identify the two vowels as a function of F0 difference was measured. Total correct scores for correctly identifying both the vowels or at least a single correct vowel were calculated. Since most of the participants failed to identify both the vowels correctly, single correct scores were considered for further evaluation. Total correct scores were significantly poorer for children with RD than TD children, suggesting that children with RD exhibit poor stream segregation ability. The perception of concurrent vowels depends on two cues, that is, F0 and formant difference. When the two vowels differ in F0, phase-locking for these F0 cues plays a significant role in segregating both the vowels ^48^. However, when the F0 is the same, phase-locking to the formant difference plays a vital role in differentiating the two vowels. Phase locking for the F0 and formant difference depends on the TFS sensitivity. Results revealed that children with RD have poorer TFS sensitivity than TD children. Poor TFS sensitivity could be because of the weak phase-locking ability in children with RD. The poor performance of children with RD in the present study supports the hypothesis that, any deviant recognition of fast acoustic events in the speech hampers the normal development of phonological systems ^17^. The poor temporal auditory functioning in children with dyslexia has been linked to the sluggish attentional shifting hypothesis. The children with reading disabilities demonstrate slower shifting of attention in a situation requiring to automatically engage and disengage the attentional focus from one stimulus to the next, like in rapid auditory tone sequences or in speech streams ^46^.

## Conclusion

Based on the current findings, it can be concluded that children with RD show impairment in auditory stream segregation as they performed significantly poorer than TD children on both TFS and concurrent vowel identification measures. The outcome of the current study helps to understand the underlying mechanism responsible for speech perception deficits seen in children with reading disabilities. Results of the current study will lead to further research on developing rehabilitation strategies and assistive device selection.

## Data availability

### Underlying data

Harvard Dataverse: Temporal processing data. https://doi.org/10.7910/DVN/XGTMAJ.

File ‘Temporal fine structure sensitivity (TFS) and Concurrent Vowel Perception in Reading disability’ contains TFS and CVP scores for all children included in this study.
