# Supplementary material for: Comparison of temporal fine structure sensitivity and concurrent vowel perception between children with and without reading disability
Source: F1000Res. 2021 Dec 15;9:1271. Originally published 2020 Oct 23. [Version 2] doi: 10.12688/f1000research.21544.2 (PMC8729192; doi:10.12688/f1000research.21544.2)
Supplement: Peer review history

# Peer review: Reviewer response for version 2

**DOI:** 10.5256/f1000research.78081.r115345

**Published:** 2022-02-02

**Authors:** Jayakumar Thirunavukkarasu, Vijaya Kumar Narne

**Affiliations:** 1 Associate Prof. in Speech Sciences, Department of Speech-Language Sciences, All India Institute of Speech and Hearing, Mysore, Karnataka, India; 2 IIT Karpur, Kanpur, India

The reviewers addressed all the questions raised by us in the previous version. However, this statement is difficult to follow, authors need explicitly state the same: **“**Since the children could not identify both the vowels, single correct scores were considered”.

Is the work clearly and accurately presented and does it cite the current literature?

Partly

If applicable, is the statistical analysis and its interpretation appropriate?

Partly

Are all the source data underlying the results available to ensure full reproducibility?

Partly

Is the study design appropriate and is the work technically sound?

Yes

Are the conclusions drawn adequately supported by the results?

Yes

Are sufficient details of methods and analysis provided to allow replication by others?

Yes

Reviewer Expertise:

Speech production and perception, Voice Sciences, Event-related potentials

We confirm that we have read this submission and believe that we have an appropriate level of expertise to confirm that it is of an acceptable scientific standard.

# Peer review: Reviewer response for version 2

**DOI:** 10.5256/f1000research.78081.r115346

**Published:** 2022-01-04

**Authors:** Ramesh Kaipa

**Affiliations:** 1 Department of Communication Sciences and Disorders, Oklahoma State University, Stillwater, OK, USA

Thank you to the authors for addressing my comments. This is an interesting and timely paper that would promote the readership of F1000.

Is the work clearly and accurately presented and does it cite the current literature?

Partly

If applicable, is the statistical analysis and its interpretation appropriate?

Yes

Are all the source data underlying the results available to ensure full reproducibility?

Yes

Is the study design appropriate and is the work technically sound?

Yes

Are the conclusions drawn adequately supported by the results?

Yes

Are sufficient details of methods and analysis provided to allow replication by others?

Partly

Reviewer Expertise:

Speech and Language Intervention

I confirm that I have read this submission and believe that I have an appropriate level of expertise to confirm that it is of an acceptable scientific standard.

# Peer review: Reviewer response for version 1

**DOI:** 10.5256/f1000research.23738.r84199

**Published:** 2021-06-21

**Authors:** Ramesh Kaipa

**Affiliations:** 1 Department of Communication Sciences and Disorders, Oklahoma State University, Stillwater, OK, USA

In the current study, the authors have attempted to investigate temporal fine structure in children with reading disability. It is an interesting and timely study but I have some minor comments to improve the rigor of this study.

I would appreciate it if the authors are able to provide some information on tests that investigate TFS.
Is Reading Acquisition Profile in Kannada a test for reading disability? 
Was it not possible for the authors to use a standardized test such as Dyslexia Assessment for Languages in India (DALI) to assess for reading disability instead of using the 2SD approach?
I was curious as to why did the authors use synthesized vowels as stimuli for the vowel identification task over live voice?
5. Although the authors mention that they intended to correlate the outcomes of the vowel identification task and the TFS1, I failed to notice the results of the correlation.

Is the work clearly and accurately presented and does it cite the current literature?

Partly

If applicable, is the statistical analysis and its interpretation appropriate?

Yes

Are all the source data underlying the results available to ensure full reproducibility?

Yes

Is the study design appropriate and is the work technically sound?

Yes

Are the conclusions drawn adequately supported by the results?

Yes

Are sufficient details of methods and analysis provided to allow replication by others?

Partly

Reviewer Expertise:

Speech and Language Intervention

I confirm that I have read this submission and believe that I have an appropriate level of expertise to confirm that it is of an acceptable scientific standard, however I have significant reservations, as outlined above.

# Response

**Published:** 2021-11-25

**Authors:** Somashekara Haralakatta Shivananjappa

**Affiliations:** som.shekar@manipal.edu, India

We thank the reviewer for the comments

**Comment 1: **I would appreciate it if the authors are able to provide some information on tests that investigate TFS.

**Response 1:** Thank you. The tests for measuring TFS has been mentioned in the introduction section. Due to the word limit, the detailed description of each test has not been mentioned. However, the relevant literature has been cited for the readers to explore further. Page No. 4 (Line no. 14-16)

**Comment 2:** Is Reading Acquisition Profile in Kannada a test for reading disability?

**Response 2:** Though the Reading Acquisition Profile in Kannada is not a test to identify reading disability, it is a content validated reading assessment battery to profile the language skills, metaphonologogical skills, reading and writing skills, reading comprehension tests, and listening comprehension that are key determiners of reading disability in children. The children’s performance on RAP-K supplements the identification of reading disability. It is developed for children learning to read Kannada, following the curriculum affiliated to the Karnataka state board, which is similar to the present study population. Hence, considering the 2SD approach was found to be feasible.

 Comment 3: Was it not possible for the authors to use a standardized test such as Dyslexia Assessment for Languages in India (DALI) to assess for reading disability instead of using the 2SD approach?

 Response 3: Yes. DALI could have also been used. However, DALI was not available in the center at the time of the study.

 Comment 4: I was curious as to why did the authors use synthesized vowels as stimuli for the vowel identification task over live voice?

 Response 4: In the current study synthesized vowels were preferred over the naturally produced vowels to exert better control over the pitch contours and formants.

 Comment 5: Although the authors mention that they intended to correlate the outcomes of the vowel identification task and the TFS1, I failed to notice the results of the correlation.

 Response 5: Thanks for pointing out the typological error, and the sentence has been removed from the manuscript.

# Peer review: Reviewer response for version 1

**DOI:** 10.5256/f1000research.23738.r84193

**Published:** 2021-05-24

**Authors:** Prashanth Prabhu P

**Affiliations:** 1 Department of Audiology, All India Institute of Speech and Hearing, Mysuru, India

The authors have attempted to assess the temporal fine structure and concurrent vowel perception in those with reading disabilities. The article is written well but has the following issues to be addressed:

- The authors should clarify the difference between dyslexia and reading disability (RD) as the authors use RD throughout the manuscript.
- It is not clear how writing difficulties, cognitive delays, auditory processing disorder was ruled out in the participants. This is important as it can affect the test results.
- How did the authors decide that the participants did not have any gross otologic and neurologic deficits?
- Which test was used to determine the normality of the data?
- The authors carried out repeated measures ANOVA and post hoc comparisons. The results of post-hoc comparisons are missing. The subsequent discussion on the same should be added.
- The authors also report that they carried out ‘Pearson correlation co-efficient to know the association between the performance of TFSI with concurrent vowel identification abilities among RD and TD’. However, the results of the above are completely missing.
- The major content in the last paragraph is a repetition of the results which is not necessary.
- Discussion should be further strengthened on reasons for poor performance and its clinical implications.
- Limitations of the study should be addressed.

Is the work clearly and accurately presented and does it cite the current literature?

Partly

If applicable, is the statistical analysis and its interpretation appropriate?

Partly

Are all the source data underlying the results available to ensure full reproducibility?

Yes

Is the study design appropriate and is the work technically sound?

Partly

Are the conclusions drawn adequately supported by the results?

Partly

Are sufficient details of methods and analysis provided to allow replication by others?

Partly

Reviewer Expertise:

Psychoacoustics, Tinnitus and Hyperacusis, Auditory neuropathy spectrum disorders, central auditory processing disorders

I confirm that I have read this submission and believe that I have an appropriate level of expertise to confirm that it is of an acceptable scientific standard, however I have significant reservations, as outlined above.

# Response

**Published:** 2021-11-25

**Authors:** Somashekara Haralakatta Shivananjappa

**Affiliations:** som.shekar@manipal.edu, India

We thank the reviewer for the comments. 

 Following are the responses to the reviewer's comments. 

**Comment 1:** The authors should clarify the difference between dyslexia and reading disability (RD) as the authors use RD throughout the manuscript.

**Response 1: **Incorporated in the first paragraph of the introduction.  Page No. 3 (Line no. 4-9)

**Comment 2: **It is not clear how writing difficulties, cognitive delays, auditory processing disorder was ruled out in the participants. This is important as it can affect the test results.

**Response 2: **The present study concentrated on children with reading disabilities alone, hence no writing difficulties were not assessed. Children from both groups belong to schools for typically developing children and had age-appropriate language skills according to Linguistic Profile Test (LPT). Since the study aimed to assess auditory processing skills of children with RD in comparison with typically developing children using TFS and concurrent vowel identification, the auditory processing was not separately assessed.

**Comment 3: **How did the authors decide that the participants did not have any gross otologic and neurologic deficits?

**Response 3: **All the children were evaluated for hearing sensitivity using pure tone audiometry. The parental interview was conducted to determine the history of otological diseases and neurological deficits.

**Comment 4:** Which test was used to determine the normality of the data?

**Response 4: **Shapiro-Wilk was used to determine the normality of the data.

**Comment 5: **The authors carried out repeated measures ANOVA and post hoc comparisons. The results of post-hoc comparisons are missing. The subsequent discussion on the same should be added **.**

**Response 5: **ANOVA with repeated measures revealed a significant main effect of reading ability on CVI scores. Also, the effect of f0 difference was significant. Nevertheless, the interaction between reading ability and F0 difference was not significant. Therefore, the follow-up pair-wise comparison was not performed, as the primary objective of the study was to investigate the effect of reading ability on CVI. However, to comply with the reviewers’ suggestion, we have now performed an independent sample ‘t’ test to assess the effect of reading ability on CVI at each F0 difference condition. The findings are mentioned in the results section. Page No.8 (Line no. 1-5), and Page No. 9 (Line no.13 -23).

**Comment 6:**The authors also report that they carried out ‘Pearson correlation co-efficient to know the association between the performance of TFSI with concurrent vowel identification abilities among RD and TD’. However, the results of the above are completely missing.

**Response 6: **Thanks for pointing out the typological error, and the sentence has been removed from the manuscript. Page No. 6 (Line No. 38-40).

**Comment 7: **The major content in the last paragraph is a repetition of the results which is not necessary

**Response: **Incorporated.

**Comment 8: **Discussion should be further strengthened on reasons for poor performance and its clinical implications.

**Response 8: **Incorporated. Page No. 8 (Line No. 29-34).

**Comment 9: **Limitations of the study should be addressed.

**Response 9: **Incorporated. Page No.8 (Line No. 35-38)

# Peer review: Reviewer response for version 1

**DOI:** 10.5256/f1000research.23738.r75988

**Published:** 2021-02-08

**Authors:** Jayakumar Thirunavukkarasu, Vijaya Kumar Narne

**Affiliations:** 1 IIT Karpur, Kanpur, India; 2 Associate Prof. in Speech Sciences, Department of Speech-Language Sciences, All India Institute of Speech and Hearing, Mysore, Karnataka, India

The manuscript studies the temporal fine structure and concurrent vowel perception in normal-hearing and Dyslexic children. The manuscript is well written with sufficient review and the methods employed are appropriate. I have some major issues and minor comments regarding the current version of manuscript.

**Major comments:** 

1. Introduction largely concentrates on studies from USA/EU data, not much data from Indian. In our view, it is important as the phoneme-graphic correspondence is significantly different from English. Further, auditory processing impairment is correlated with VOT from western data. This cannot be directly implied for the same in the Indian population as VOT properties of Indians are very different. So, I feel that Introduction needs more specific to the population under study. 
2.  Are the results of the TFS-1-test reliable for the age range under study? because the TFS values are poor compare to the literature. From the description in the present study, it is difficult to understand if the results are reliable. Further, there is a published study cited or that available none adopted TFS-1 test many adopted TFS-LF. This needs to be clarified.
3. The concurrent vowel identification task looks simple discrimination task, and it is far from auditory stream segregation. The author needs to provide evidence that such a task is considered as auditory stream segregation. 

**Minor Comments:**

1. Introduction-Para-1: Authors provide the % of RD from the western population. It would be more appropriate if they provide the same from the Indian population.
2. Introduction-Para-1: Impaired phoneme-grapheme correspondence impairment varies largely with Language. As English is complicated with reference to phoneme-grapheme correspondence, compare to Indian languages. 
3. Method-TSF-test: Did the authors adopted the procedure developed by Prof. Moore or they used software developed by them. It is not clear from the description. 

Is the work clearly and accurately presented and does it cite the current literature?

Partly

If applicable, is the statistical analysis and its interpretation appropriate?

Partly

Are all the source data underlying the results available to ensure full reproducibility?

Partly

Is the study design appropriate and is the work technically sound?

Yes

Are the conclusions drawn adequately supported by the results?

Yes

Are sufficient details of methods and analysis provided to allow replication by others?

Yes

Reviewer Expertise:

Speech production and perception, Voice Sciences, Event-related potentials

We confirm that we have read this submission and believe that we have an appropriate level of expertise to confirm that it is of an acceptable scientific standard, however we have significant reservations, as outlined above.

# Response

**Published:** 2021-11-25

**Authors:** Somashekara Haralakatta Shivananjappa

**Affiliations:** som.shekar@manipal.edu, India

We thank the reviewers for the comments. 

 Following are the response to the reviewer's comments. 

**Comment 1:** Introduction largely concentrates on studies from USA/EU data, not much data from Indian. In our view, it is important as the phoneme-graphic correspondence is significantly different from English. Further, auditory processing impairment is correlated with VOT from western data. This cannot be directly implied for the same in the Indian population as VOT properties of Indians are very different. So, I feel that Introduction needs more specific to the population under study.

**Response 1:** Thanks for the comment. The issue has been addressed in the response to the fifth comment.

**Comment 2:** Are the results of the TFS-1-test reliable for the age range under study? because the TFS values are poor compare to the literature. From the description in the present study, it is difficult to understand if the results are reliable. Further, there is a published study cited or that available none adopted TFS-1 test many adopted TFS-LF. This needs to be clarified.

**Response 2: **We do agree that the TFS values are poor compare to the literature. We would like to bring to the reviewer’s notice that, the primary objective of the study is to compare the performance between the typically developing children and children with RD. The test was performed under the assumption that the difficulty level would be the same for both groups. Also, participants of both groups were underwent testing after the training with the task. We do agree with the reviewer that TFS-LF is another test to assess the temporal fine structure sensitivity. However, various studies have established that discrimination of harmonic complex and frequency-shifted in-harmonic complex (as in TFS1 test) can be used as a measure of TFS sensitivity. E.g., (Marmel et al., 2015; Moore & Sek, 2009; Moore & Sęk, 2011). 

**Response 3: **Incorporated. Page No. 3 (Line no. 2-6)

**Comment 3: **The concurrent vowel identification task looks simple discrimination task, and it is far from auditory stream segregation. The author needs to provide evidence that such a task is considered as auditory stream segregation.

**Response 3: **Various studies(Cheveigné, 1997; Chintanpalli et al., 2016; Settibhaktini et al., 2021; Settibhaktini & Chintanpalli, 2020; Smith et al., 2018) have indicated that F0 guided segregation as a mechanism behind concurrent vowel perception. Hence in the current study, concurrent vowel identification task is used as a measure of concurrent sound segregation. Each vowel acts as an interferer for the other vowel, thereby increasing the perceptual difficulty. For the optimal identification, the target vowel should be segregated from the noise for which the F0 difference is one of the potential cues.

**Comment 4: **Introduction-Para-1: Authors provide the % of RD from the western population. It would be more appropriate if they provide the same from the Indian population.

**Response 4: **Incorporated. Page No. 3 (Line no. 2-6)

**Comment 5: **Introduction-Para-1: Impaired phoneme-grapheme correspondence impairment varies largely with Language. As English is complicated with reference to phoneme-grapheme correspondence, compare to Indian languages.

**Response 5: **The languages vary depending upon the orthographic transparency and consistency. The alphabetic language such as English is less transparent and inconsistent because one grapheme (alphabet) can correspond with many phonemic realizations. In contrast, alphasyllabary languages are more transparent and consistent, because one grapheme (Akshara) corresponds to single-syllable most of the time. To be specific, the primary mapping of phonology in Kannada is at the level of the orthographic syllable (syllabic) but the symbols of the language also embody phoneme markers (hence alphasyllabic). Children with dyslexia in alphasyllabary languages exhibit primary deficits in the acquisition of Akshara knowledge besides phonological awareness deficits at the syllable level. Hence, the basic word decoding is universal across all the languages, where the sounding out requires the conversion of the written symbol (grapheme) with the corresponding linguistic unit (phoneme or syllable), which necessitates both phonological and orthographic processing.

**Comment 6: **Method-TSF-test: Did the authors adopted the procedure developed by Prof. Moore or they used software developed by them. It is not clear from the description.

**Response 6: **The TFS-test developed by Prof. Moore was not used in the current study. However, a similar test was adopted. The procedure used in the current study was similar to the test developed by Prof. Moore, except for the background noise. Pink noise was used to prevent the audibility of combination tone and spectral cues, instead of TEN noise. The correction is incorporated in the revised manuscript.
